# Dual Plating in Bicondylar Proximal Tibia Fractures and Its Functional Outcomes

**DOI:** 10.7759/cureus.103803

**Published:** 2026-02-17

**Authors:** Yogesh Bhangale, Kiran Wandile, Supratim Roy, Bhavesh Patidar, Shreyas Rekhate, Chandrashekhar M Badole

**Affiliations:** 1 Orthopaedics, Mahatma Gandhi Institute of Medical Sciences, Sewagram, Wardha, IND

**Keywords:** bi-column plating, bicondylar, bicondylar fracture, dual incision, dual plating, fracture, schatzker type, schatzker types, tibial plateau, tibial plateau fracture

## Abstract

Background: Managing such fractures is a difficult challenge in developing countries. Many kinds of internal fixation devices, including recently developed plates with screws, have been applied to treat these complex fractures. In this study, dual plating of bicondylar proximal tibia fractures is done to assess the outcome of the patients in terms of knee range of motion, fracture union, and reduction, which dictates the functional outcome.

Materials and methods: A prospective study was carried out to assess the outcomes of proximal tibial fractures in the department of orthopaedics from May 2022 to April 2025. A total of 30 patients with bicondylar proximal tibia fractures were operated on. The patients were monitored for an average of six months. Data were entered into a spreadsheet and analyzed using descriptive statistics. Continuous variables were expressed as mean ± standard deviation, while categorical variables were summarized as frequencies and percentages.

Result: Mean time to fracture union was 3.81 ± 0.82 months. The mean range of motion of the knee joint was 134.5 ± 11.09 degrees (range: 0° to 10° for extension and range: 0° to 120°-150° for flexion). At the last follow-up, the mean Rasmussen's functional grading score was 27.96 ± 2.95. Five (16.67)% patients in the current study experienced complications.

Conclusion: Rigid and stable fixation with proper articular reduction, maintaining the soft tissue integrity, is the most important determinant for outcome in the treatment of Schatzker V and VI proximal tibia fractures, which can be achieved with bi-column fixation with locking plates using the dual approach.

## Introduction

Proximal tibial plateau fractures are the most common weight-bearing joint fractures [1], accounting for approximately 1% of all fractures and 8% of fractures in the elderly [2]. Such injuries vary widely, ranging from direct or indirect mechanisms with low-velocity trauma to high-velocity trauma. The proximal tibia and surrounding soft tissue absorb a significant amount of energy from direct injuries, which include dashboard injuries, bumper fractures, falls from a height, and motorcycle accidents. This can result in significant tibia fractures and comminution, as well as serious soft tissue injuries. Axial compression caused by a fall, severe twisting, or an intense angular motion that occurs in high-energy sports injuries (such as skiing, football, rugby, etc.) are examples of an indirect mechanisms that result in high-energy proximal tibia fractures [3].

Congruency, stability, optimum load distribution, and adequate cartilage biological activity are all essential for optimal joint performance. Every intraarticular fracture entails the restoration of these characteristics. Slight articular mismatch up to 2 mm is acceptable or otherwise compromises the functional ability of the lower limb; therefore, rigid and stable fixation is of paramount importance. Open reduction and visualization of the articular fragment aid in accurate reduction, and early rehabilitation is possible only with stable fixation [4].

Preoperative planning is crucial in managing such fractures. Evaluation of soft tissue injuries around the knee is essential. Various factors are important for planning the treatment of such fractures, like comminution at the fracture site, articular impaction, and associated soft tissue injuries, which determine the timing of surgery, approach, and implants [5]. A range of treatment options for tibial plateau fractures includes traction, casts or braces, hybrid, ring, or AO fixators, limb reconstruction system, locking plates, column fixations, single incision, dual incision or minimally invasive percutaneous plate osteosynthesis (MIPPO), and recently developed techniques like arthroscope-assisted fracture reduction and fixation with implants [6].

Medial plateau fractures with neurovascular and soft tissue injuries, along with increased load on the medial articular surface during weight bearing, causing varus collapse, have been reported in the literature, leading to poor prognosis. Thus, such fractures require a stable and anatomic reduction under direct visualization through a medial or posteromedial approach [7]. Bi-column fixation of the proximal tibia using separate incisions with locking plates tends to have lower subsidence and wound complications [8,9]. Whereas, the use of a single incision increases wound complications due to increased damage to compromised soft tissue, periosteal stripping, and devitalization, impacting fracture healing and elevating the risk of wound infection [10].

Hence, this study aims to determine the functional outcome of bi-column plating in Schatzker type V and VI of tibial plateau fractures. Despite the availability of multiple fixation strategies for bicondylar tibial plateau fractures, there remains limited prospective evidence evaluating functional outcomes following bi-column fixation using dual locking plates, particularly in high-energy Schatzker type V and VI fractures. Most available studies are retrospective, heterogeneous in methodology, or originate from high-resource settings, with relatively fewer studies focusing on functional recovery in rural and resource-constrained environments where delayed presentation and compromised soft tissue conditions are common. Therefore, the present study was undertaken to address this gap by prospectively evaluating the functional outcomes, fracture union, and complications following dual plate fixation of bicondylar tibial plateau fractures in a rural tertiary care setting.

## Materials and methods

A prospective interventional follow-up study was conducted in the rural medical centre in the Department of Orthopaedics, Mahatma Gandhi Institute of Medical Sciences (MGIMS), Sewagram, Wardha, India between May 2022 and April 2025. The initial data for this study were originally collected as part of a continuous institutional quality-improvement database for proximal tibia fractures. Once the decision was made to analyze this cohort for formal publication, IRB approval was sought and granted for the secondary use of this de-identified data with the approval number MGIMS/IEC/ORTH/27/2023.

Thirty cases attending the orthopaedics OPD or the accident and emergency centre with displaced tibial plateau fractures under Schatzker's type V and VI categories were part of the study. These cases required bi-column fixation using locking plates with an anterolateral and a medial or posteromedial approach. The sample size of 30 was determined using a feasibility-based approach as the study was designed with a fixed recruitment period. All eligible patients presenting during this period were enrolled, resulting in a final sample of 30 cases.

Patient recruitment was conducted using a non-probability consecutive sampling method, in which all patients aged >18 years with radiologically confirmed displaced bicondylar tibial plateau fractures who met the inclusion and exclusion criteria and provided informed consent were enrolled consecutively. This approach helped minimize selection bias and reflect real-world clinical practice.

Patient inclusion and exclusion criteria

Under this study, patients met the following essential criteria: they were adults aged 18 years or older and had a confirmed radiological diagnosis of a displaced bicondylar tibial fracture classified as Schatzker type V or VI [5]. All participants demonstrated their willingness to participate by providing written informed consent.

Patients were excluded if they had open (compound) bicondylar proximal tibial fractures, pathological fractures, prior surgery around the knee, or associated neurovascular injury or compartment syndrome. In addition, polytrauma patients were not eligible if they had head injury, spinal injury, pelvic fractures, or any other fractures involving the ipsilateral or contralateral lower limb.

Preoperative protocol

The mode of injury and the severity of the trauma were assessed from the history of the patient. All patients were thoroughly evaluated and carefully examined for deformity, swelling, ecchymoses, blisters, status of skin conditions, signs of compartment syndrome, and distal neurovascular deficits. The patient's general condition was stabilized. Patients received appropriate analgesia, and the affected lower limb was immobilized with either a long leg slab or skeletal traction, depending on the degree of comminution. The limb was elevated using a Böhler-Braun frame or splint until soft-tissue swelling subsided, as indicated by the appearance of the wrinkle sign and improvement in local skin condition. All patients were given preoperative antibiotics one hour before surgery. Patients were operated on under spinal or general anesthesia, according to the choice of the anesthetist, depending on the patient's status.

In all patients, surgery was performed in the supine position, with the affected limb placed on a radiolucent wooden frame or a sterile bolster under the knee. A pneumatic tourniquet was used in all cases. Implants and instrument trolleys were arranged and checked prior to the administration of anesthesia.

Surgical Procedure

**Figure 1:**
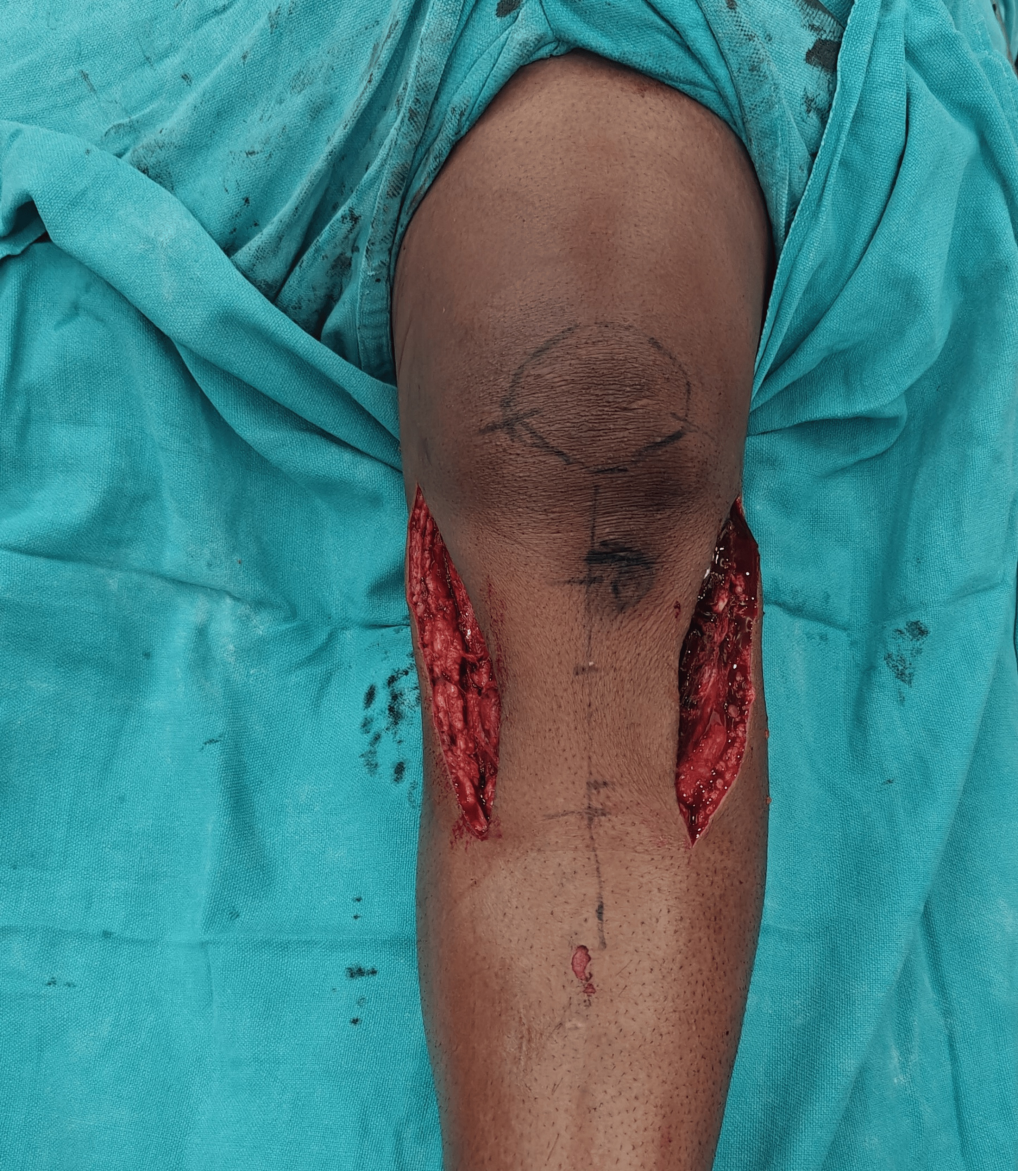
Intraoperative image showing dual incision technique used in bi-column fixation of bicondylar tibial plateau fracture.

**Figure 2:**
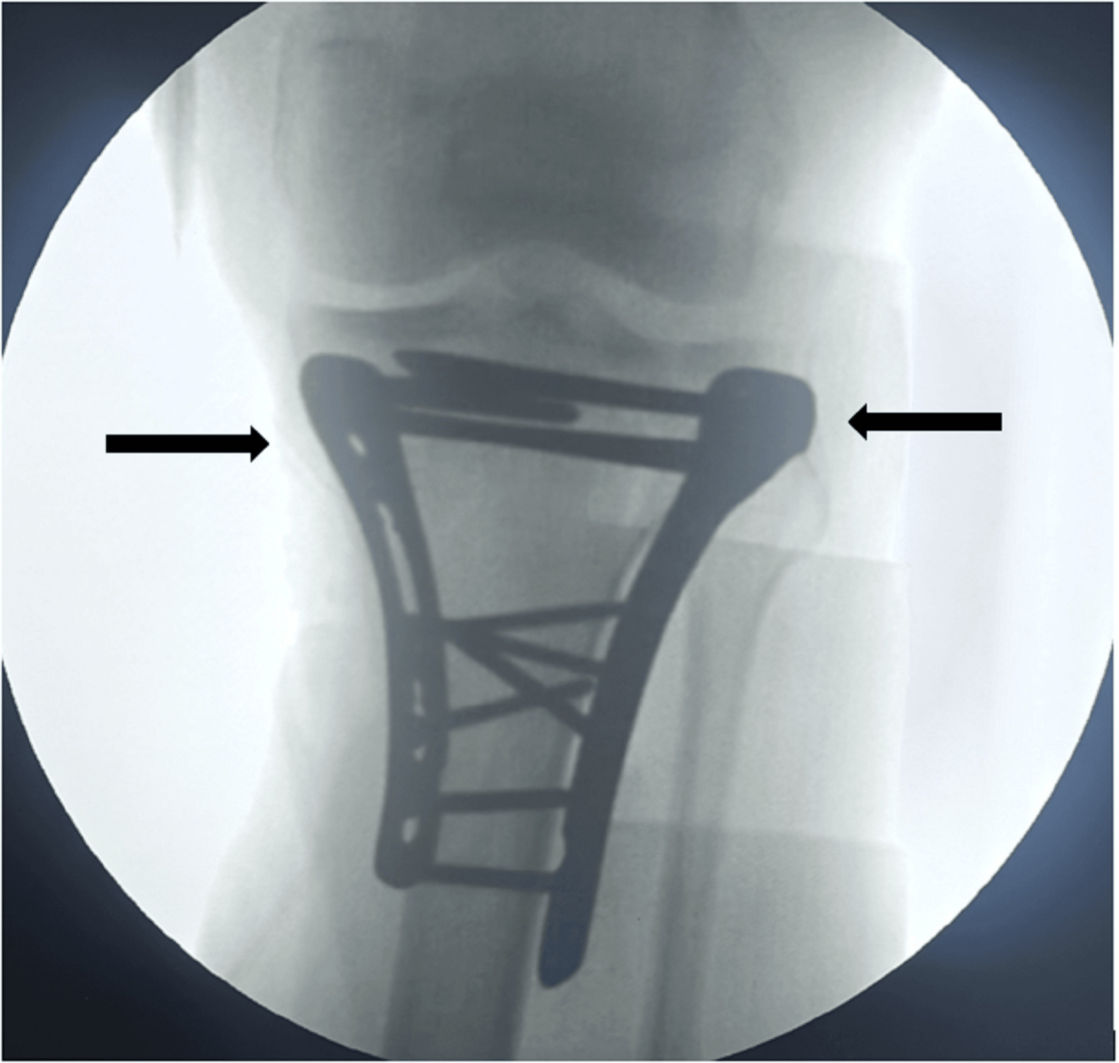
Intraoperative fluoroscopic image showing anteroposterior (AP) view of bi-columnar fixation of bicondylar proximal tibia fracture. Arrow showing acceptable reduction at articular site with bi-columnar locked plates using dual-incision approach.

**Figure 3:**
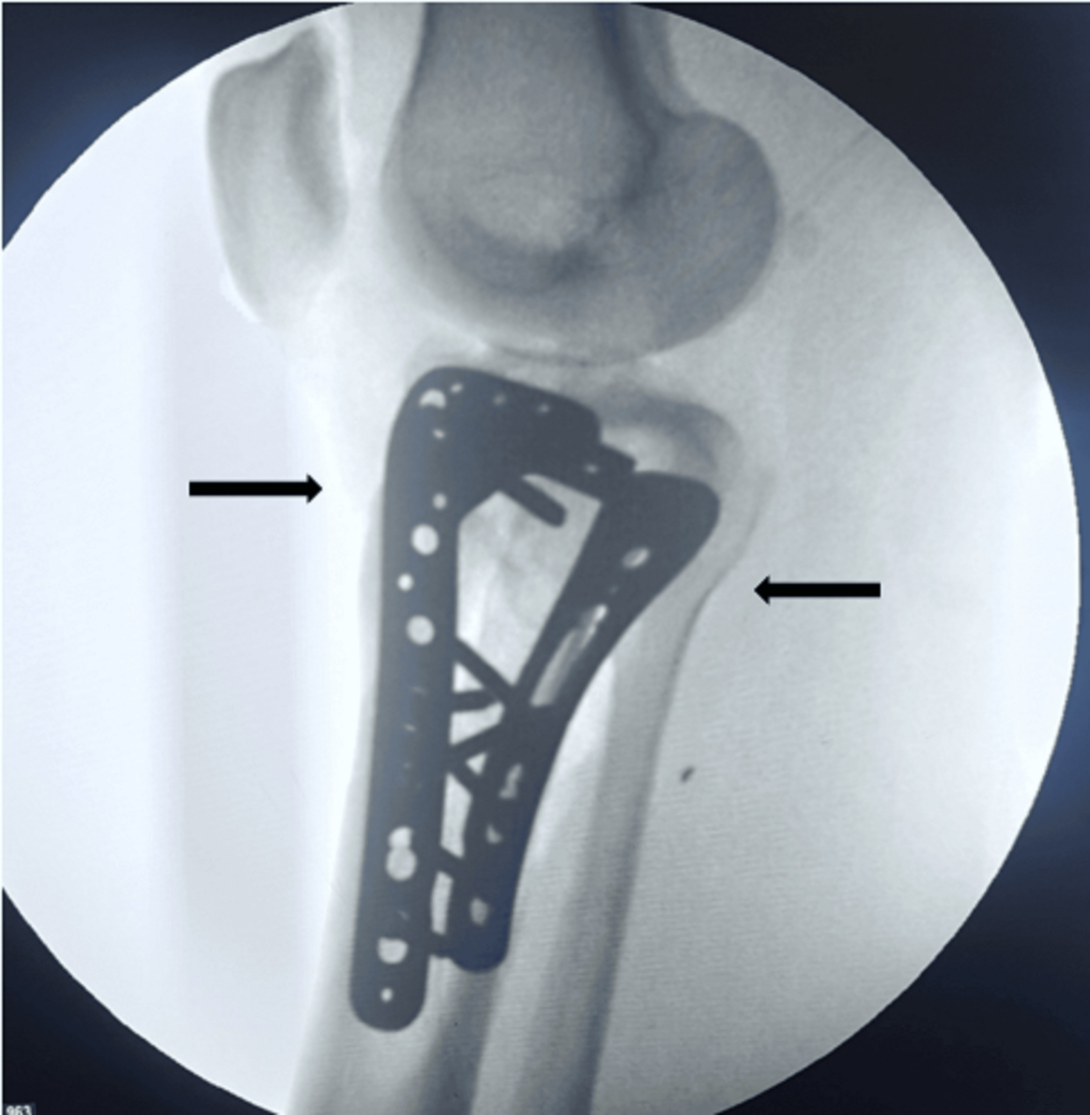
Intraoperative fluoroscopic image showing lateral view of bi-columnar fixation of bicondylar proximal tibia fracture. Arrow showing absolute stability and acceptable reduction with bi-columnar locking plates using dual-incision approach.

The surgical technique followed standard principles of dual-incision bi-column fixation for bicondylar tibial plateau fractures as described in the literature, without any modification. All surgeries were performed under fluoroscopic control with an anterolateral and medial or posteromedial approach as decided preoperatively after evaluating the fracture geometry, displacement, and articular depression on computed tomography (CT) scans (Figures 1, 2, 3). Fracture was reduced using various reduction clamps, and articular depressions were reduced using bone tamps under C-arm guidance and held temporarily with K-wires. Suitable design locking plates, like 'T' or 'L' buttress plate, raft plate, or a locking proximal tibia hockey plate was used to fix the fragment. Special attention was taken to restore the length axis and alignment. Surgery was performed under torniquet and the wound was closed in layers under a negative suction drain.

Postoperative Protocol

Wound inspection and drain removal were done on the second postoperative day. Range of motion started immediately after surgery, from postoperative day 1 and quadriceps and hamstring strengthening exercises were started. Stitches were removed at the end of two weeks after surgery. Partial weight-bearing was allowed after signs of clinical union appeared (commonly six to eight weeks). Full weight-bearing to the patient was allowed once the radiological union was achieved (commonly 12-16 weeks). The union status of the fracture was determined by the absence of pain and tenderness at the fracture site and callus formation at the fracture site on radiological assessment in both views.

Follow-up

Follow-up was conducted at six weeks, three months, and six months, or until the radiological union of the fracture. Functional outcome was assessed according to Rasmussen's clinical criteria of functional assessment [11].

Rasmussen’s functional score is based on pain, walking capacity, range of motion, stability, and extension lag, with a maximum score of 30 points. Excellent outcome (28-30 points) indicates near-normal knee function: no pain or only occasional mild pain, normal walking capacity without support, full or near-full knee range of motion, no instability of the knee joint, and no or minimal extension lag. Good outcome (24-27 points) indicates satisfactory functional recovery: mild pain after prolonged activity, slight limitation in walking or stair climbing, mild restriction of knee movements, no significant instability, minimal extension lag. Fair outcome (20-23 points) indicates functional limitation but ambulatory knee: moderate pain during daily activities, reduced walking capacity, may require support, noticeable restriction of knee movements, mild instability may be present, definite extension lag. Poor outcome (<20 points) indicates poor functional result with disability: severe or persistent pain, marked limitation of walking ability, gross restriction of knee motion, significant instability of the knee joint, and marked extension lag.

## Results

Demographics

Demographic data of the current study are as follows (Table 1).

**Table 1:** Demographic data of the study participants showing various parameters.

Parameters		No. of Patients	Percentage (%)
Age	21-30 years	4	13.33 %
	31-40 years	9	30 %
	41-50 years	6	20 %
	50-60 Years	7	23.33 %
	>60 years	4	13.33 %
Sex	Male	24	80 %
	Female	6	20 %
Mode of injury	RTA	21	70 %
	Domestic falls	8	26.67 %
	Assault	1	03.33 %
Laterality	Left	16	53.33 %
	Right	14	46.67 %
Fracture pattern	V	09	30 %
	VI	21	70 %

The mean age in this study was 44.67 ± 12.77 years ( p-value: 0.887). The time interval between the injury and definitive surgery ranged from three days to a maximum of 10 days. The average time between injury and surgery was 7 ± 2.14 days. Average blood loss was 168.2 ± 11.55 ml. It was observed that 14 patients (46.67%) had blood loss between 110 and 159 ml. The minimum time duration of surgery was 132 min, and the maximum was 192 min. The average duration of surgery was 135.6 min ± 16.7 min.

**Figure 4:**
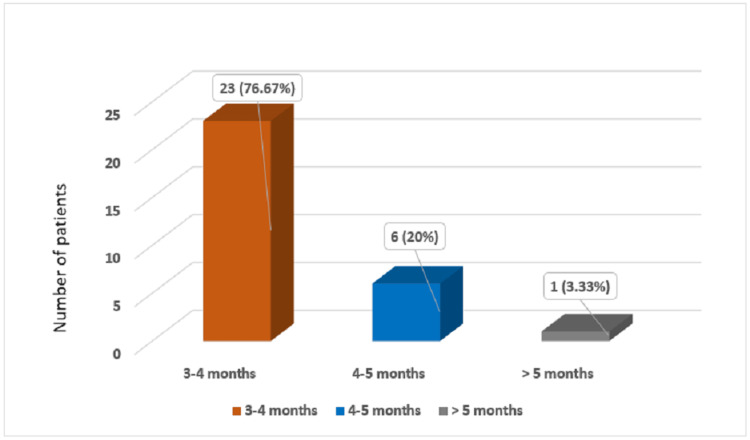
Time of fracture union showing majority of the patients had fracture union between 3-4 months.

Whereas, 23 patients (76.67%) started full weight bearing between 2.5 and 3.5 months, with a mean time to start full weight bearing ambulation of 3.15 ± 0.70 months. The majority of the patients (21, 70%) have achieved a range of motion between 130 degree to 150 degrees. The mean flexion at the knee joint was 134.5 ± 11.09 degrees. In our study, there was zero-degree extension lag in 23 (76.67%) of cases, with a mean extension lag of 1.46 ± 2.8 degrees. Maximum extension lag of 10 degrees was observed in one patient (03.33%). Mean time to fracture union was 3.81 ± 0.82 months, with most of the patients (23, 76.67%) between three and four months (p-value: 0.223). There was a single case (03.33%) of non-union (Figure 4).

**Figure 5:**
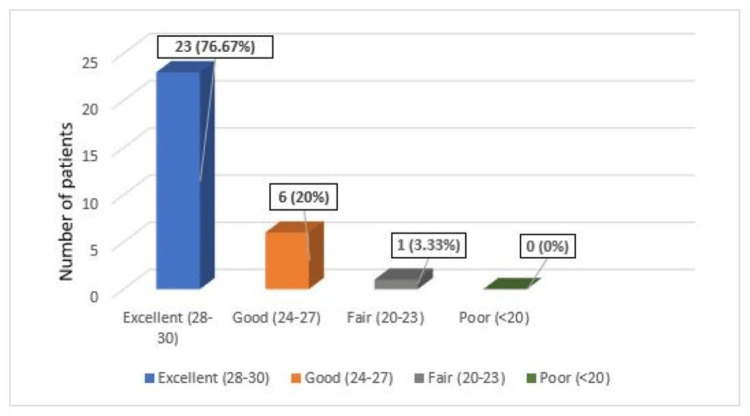
Functional outcomes of the given study showing majority had excellent outcomes.

Functional outcomes were assessed using Rasmussen's functional outcome criteria [11], with most patients having excellent to good outcomes (p-value: 0.990). The mean functional outcome was 27.96 ± 2.95 (Table 2, Figure 5).

**Table 2:** Functional outcomes among study participants according to Rasmussens criteria.

Functional outcomes	Number of patients	Percentage
Excellent (28-30)	23	76.67%
Good (24-27)	6	20%
Fair (20-23)	1	3.33%
Poor (<20)	0	0%
Total	30	100%
Mean ± SD	27.96 ± 2.95	

Complications

In our study, wound-related complications were seen in five patients (16.66%). Three patients (10%) had serous discharge, one case of non-union (3.33%) and one patient (3.33%) associated with wound gaping and implant exposure. There were no cases of knee instability.

## Discussion

Biomechanical studies indicate that dual locking plate fixation provides greater stability compared to a lateral locking plate alone [12]. Our study assessed the outcomes of such fractures treated with dual locking plates in terms of fracture union, range of motion, functional outcomes and complications. In our study, the majority of patients were male (24, 80%) and belonged to the 31-40-year age group, suggesting a male predominance and a higher incidence among young and middle-aged individuals, consistent with findings from previous studies [12-14].

In this study, the range of motion of the knee joint was with the mean value of 134.5 ± 11.09 degree and the mean extension lag at the knee joint was 1.46 ± 2.8 degrees. One patient (3.33%) has an extension lag of 10 degrees. The patient had serous discharge from the wound site, pain and was non-compliant with physiotherapy on follow-ups. A study by Bhalotia et al. [15] reported that a range of motion of more than 130° was achieved in 21 cases (87.5%), while in the remaining 3 cases (12.5%), it was between 110° and 130. Various other studies have reported a good range of motion in patients with bicondylar proximal tibia fractures after dual plating [14,16-20]. Raj et al. [14] reported the mean extension lag at the knee joint of 1.5° (range: 0°-10° for extension lag). Rajpal et al. [12] reported that 24 (60%) of cases were without extension lag, 12 (30%) with 0-10 degrees of extension lag, and 4 (10%) with >10 degrees of extension lag. Prasad et al. had reported a mean extension lag of 1.75 [18].

Table 3 represents a comparative analysis of functional outcomes assessed using the Rasmussen functional scoring system across multiple published studies and the present study. Good union rates have been reported with such fixation in various studies [12,15,21]. Delayed unions were noted in studies by Oh et al [17] and Raj et al. [14]. Non-union in such fractures with dual plating was also reported in the literature [18,22].

**Table 3:** Various studies with functional outcome and average mean score and its comparison with the given study.

Sr No	Study name	Scoring system	Outcome		Average mean functional score
1	Rajpal et al [12]	Rasmussen functional score	Excellent (28-30)	24 (60%)	25.85 ± 4.83
			Good (24-27)	11 (27.5%)	
			Fair (20-23)	4 (10%)	
			Poor (<20)	1 (2.5%)	
2	Raj M. et al [14]	Rasmussen functional score	Excellent (28-30)	15 (50%)	23.75 (19-28)
			Good (24-27)	12 (40%)	
			Fair (20-23)	2 (6.67%)	
			Poor (<20)	1 (3.33%)	
3	Shrestha et al [23]	Rasmussen functional score	Excellent (28-30)	15 (46.87%)	27.34 (22-30)
			Good (24-27)	13 (40.62%)	
			Fair (20-23)	3(9.37%)	
			Poor (<20)	1(3.12%)	
5	Oh et al [17]	Rasmussen functional score	Excellent (28-30)	18 (78.26%)	26 (14-30)
			Good (24-27)	3 (13.04%)	
			Fair (20-23)	2 (08.69%)	
			Poor (<20)	0 (0%)	
6	Our study	Rasmussen functional score	Excellent (28-30)	23 (76.67%)	27.96 ± 2.95
			Good (24-27)	6 (20%)	
			Fair (20-23)	1 (3.33%)	
			Poor (<20)	0 (0%)	

In the study by Rajpal et al., 60% of patients achieved excellent outcomes, with an additional 27.5% demonstrating good results, yielding a combined satisfactory outcome (excellent + good) of 87.5% [12]. Similarly, Raj et al. reported excellent and good outcomes in 50% and 40% of patients, respectively, with only a small proportion (10%) falling into the fair and poor categories [14]. Shrestha et al. also reported comparable findings, with 46.87% excellent and 40.62% good outcomes, reflecting a satisfactory outcome rate of nearly 88% [23]. These studies highlight moderate variability in mean functional scores, likely influenced by differences in fracture severity, fixation techniques, rehabilitation protocols, and duration of follow-up.

Notably, Oh et al. demonstrated a higher proportion of excellent outcomes (78.26%) and no poor results, suggesting that meticulous surgical technique and stable fixation can significantly enhance functional recovery [17]. The mean functional scores reported in these studies generally ranged between the mid-20s and high-20s, reinforcing the effectiveness of operative intervention for complex tibial plateau fractures.

In comparison, the present study shows one of the most favorable outcome distributions, with 23 (76.67%) patients achieving excellent results and 20% achieving good results, resulting in an overall satisfactory outcome rate of 96.67%. Twenty-nine patients with the given fracture healed within a period of six months, with the majority of the fractures, 23 healed in a period of 3-4 months.

Only one patient (3.33%) had a fair outcome with non-union, which was later treated with bone grafting and revision of fixation and no poor outcomes were observed. The mean Rasmussen functional score of 27.96 ± 2.95 in the present study is higher than or comparable to those reported in earlier studies, indicating superior functional recovery. This may be attributed to standardized dual-plate fixation, careful restoration of articular congruity, adherence to soft tissue principles, and early postoperative mobilization. The comparatively higher excellent outcome rate and absence of poor results in the present study further support the efficacy and reliability of this surgical approach when combined with appropriate patient selection and structured rehabilitation protocols.

Collectively, the table demonstrates a consistently favorable functional outcome following surgical management of bicondylar tibial plateau fractures, with the majority of patients in all studies achieving excellent to good results.

Complications

Table 4 provides a comparative overview of the complication profiles reported across various published studies evaluating surgical management of bicondylar tibial plateau fractures, along with the findings from the present study.

**Table 4:** Various studies showing complications and their comparison with the given study.

Sr no	Study name	Total No. of cases analysed	Complications	Number of cases	Percentage
1	Ozkaya and Parmaksizoglu [24]	22	Superficial infection	3	13.64%
			Deep infection	1	4.54%
			Articular depression	2	9.09%
2	Rohra et al [21]	34	Knee stiffness	3	8.82%
			Superficial infection	2	5.88%
			Extension lag	1	2.94%
			Loss of reduction	5	14.70%
3	Parikh et al [13]	30	Superficial infection	5	16.67%
			Deep infection	1	3.33%
			Non union	1	3.33%
			Knee instability	1	3.33%
4	Rajpal et al [12]	40	Superficial infection	1	2.5%
			Deep infection	1	2.5%
			Knee stiffness	2	5%
			Malalignment	1	2.5%
			Implant prominence	1	2.5%
5	Raj M. et al [14]	30	Superficial infection	3	10%
			Deep infection	1	3.33%
			Articular depression	1	3.33%
			Malalignment	1	3.33%
			Knee stiffness	1	3.33%
6	Bhalotia et al [15]		Infection	1	4.15%
			Malunion	1	4.15%
7	Hassankhani et al [25]	22	Infection	2	9%
			Changed work	2	9%
			Significant pain	2	9%
			Noticeable limp	2	9%
			Loss of knee function >15 days	3	13.6%
8	Olivero and MC [26]	50	Persistent pain	20	40%
			Superficial infection	5	10%
			Deep infection	2	4%
			OA changes	32	64%
			Fair limited ROM	14	28%
			Malalignment	4	8%
9	Oh et al [17]	23	Shortening of 1 cm	1	4.34%
			Screw loosening	1	4.34%
			Varus deformity	2	8.69%
			Superficial infection	1	4.34%
			Implant prominence	4	17.39%
11	Our study	30	superficial wound discharge	3	10%
			non-union	1	3.33%
			wound gaping and implant prominence	1	3.33%

Overall, the table highlights that although operative treatment of these complex fractures is associated with a range of complications, the majority are minor, manageable, and occur at relatively low frequencies.

Infection-related complications were the most commonly reported adverse events across studies. Superficial infections ranged from as low as 2.5% in the study by Rajpal et al. [12] to as high as 16.67% in the series by Parikh et al. [13], while deep infections were comparatively infrequent, generally remaining below 5% in most reports. Ozkaya and Parmaksizoglu [24] reported both superficial (13.64%) and deep infections (4.54%), emphasizing the vulnerability of soft tissues in high-energy tibial plateau fractures. Similar trends were observed in Raj et al. and Oh et al., where infection rates remained modest and were amenable to conservative or minor surgical management [14,17].

Mechanical and alignment-related complications, including loss of reduction, malalignment, articular depression, and varus deformity, were variably reported. Rohra et al. [21] documented a relatively higher rate of loss of reduction (14.7%), while articular depression was reported by both Ozkaya and Parmaksizoglu [23] and Raj et al. [14]. Malalignment and varus deformity were less frequent but clinically relevant, underscoring the importance of accurate fracture reduction, stable fixation, and meticulous intraoperative technique. Implant-related issues such as implant prominence and screw loosening were also reported, particularly in the studies by Rajpal et al. and Oh et al., reflecting challenges related to implant positioning and soft tissue coverage [12,17].

Functional complications, including knee stiffness, extension lag, persistent pain, and reduced range of motion, were reported with varying frequency. Knee stiffness was noted in several studies, including Rajpal et al., Raj et al. and Rohra et al., though generally affecting a small proportion of patients [12,14,21]. Olivero and MC reported a notably higher incidence of long-term sequelae such as persistent pain (40%), osteoarthritic changes (64%), and limited range of motion (28%), likely attributable to longer follow-up durations and inclusion of more severe fracture patterns [26].

In comparison, the present study demonstrates a relatively low complication rate, with superficial wound discharge observed in 10% of patients, non-union in 3.33%, and wound gaping with implant prominence in 3.33%. Importantly, there were no cases of deep infection, significant malalignment, or loss of reduction, suggesting that adherence to soft tissue principles, staged surgery when required, and stable dual-column fixation contributed to favorable outcomes.

Overall, the comparative data in Table 4 indicate that while complications following surgical treatment of bicondylar tibial plateau fractures are not uncommon, most are minor and manageable. The complication profile of the present study is comparable to or better than that reported in existing literature, supporting the safety and efficacy of dual-plate fixation when performed with careful patient selection, meticulous surgical technique, and structured postoperative rehabilitation.

## Conclusions

Dual plating for the treatment of bicondylar fractures of the tibia provides stable fixation, allowing for the early initiation of range of motion exercises and rehabilitation, which leads to good functional outcomes. Proper decision-making regarding the timing of the surgery, according to the soft tissue condition, planning of skin incision, soft tissue dissection, and plate placement according to the fracture anatomy, is crucial in reducing complications. When these principles are followed, dual plating can be performed safely, with a reduced risk of infection and implant-related complications, ultimately leading to improved clinical and functional outcomes.
